# Quantitative Assessment of Serine-8 Phosphorylated β-Amyloid Using MALDI-TOF Mass Spectrometry

**DOI:** 10.3390/molecules27238406

**Published:** 2022-12-01

**Authors:** Andrey A. Kuzin, Galina S. Stupnikova, Polina A. Strelnikova, Ksenia V. Danichkina, Maria I. Indeykina, Stanislav I. Pekov, Igor A. Popov

**Affiliations:** 1 Moscow Institute of Physics and Technology, 141700 Dolgoprudny, Russia; 2 Emanuel Institute of Biochemical Physics, Russian Academy of Science, 119334 Moscow, Russia; 3 Skolkovo Institute of Science and Technology, 121205 Moscow, Russia; 4 Engelhardt Institute of Molecular Biology, 119991 Moscow, Russia; 5 Siberian State Medical University, 634050 Tomsk, Russia

**Keywords:** amyloid-beta, MALDI-TOF, phosphopeptide

## Abstract

The study of the molecular mechanisms of the pathogenesis of Alzheimer’s disease (AD) is extremely important for identifying potential therapeutic targets as well as early markers. In this regard, the study of the role of post-translational modifications (PTMs) of β-amyloid (Aβ) peptides is of particular relevance. Serine-8 phosphorylated forms (pSer8-Aβ) have been shown to have an increased aggregation capacity and may reflect the severity of amyloidosis. Here, an approach for quantitative assessment of pSer8-Aβ based on matrix-assisted laser desorption/ionization time-of-flight mass spectrometry (MALDI-TOF MS) is proposed. The relative fraction of pSer8-Aβ was estimated in the total Aβ-pool with a detection limit of 1 fmol for pSer8-Aβ (1–16) and an accuracy of 2% for measurements in the reflectron mode. The sensitivity of the developed method is suitable for determining the proportion of phosphorylated peptides in biological samples.

## 1. Introduction

Alzheimer’s disease (AD) is a neurodegenerative disorder responsible for 60–80% [1] of all cases of dementia among the elderly, and is associated with dendritic instability and degeneration due to loss of synapses, which gradually leads to behavioral changes, memory and mental impairment, complete disability, and failure of vital body systems [2]. The development of pathology is accompanied by intracerebral accumulation of beta-amyloid peptides, which aggregate into various structures from low molecular weight soluble oligomers to mature fibrils with a characteristic beta-sheet fold [3]. According to numerous experimental data, beta-amyloid (Aβ), isolated from tissues and biofluids of AD patients, is represented by a variety of modified forms. Post-translational modifications (PTMs) associated with AD include isomerization, pyroglutamylation, deamidation, and oxidation [4]. Recently, attention has also been focused on phosphorylated forms of Aβ (p-Aβ), as possible participants of the pathological cascade. Potential phosphorylation sites in Aβ are Ser8/26 and Tyr10. pSer8-Aβ was reported to show a greater tendency to aggregate in vitro [5]. It has also been shown that intraneuronal accumulation of phosphorylated peptides precedes plaque formation in the brains of transgenic mice (APP/PS1) [6]. pSer8-Aβ was detected by Western blotting in dispersed, membrane-associated (SDS-soluble) and plaque-containing (formic acid-soluble) fractions obtained by differential centrifugation of brain tissue homogenate [7]. Its levels were found to be significantly higher in samples obtained from AD patients compared with pre-AD and control groups. Thus, the amount of phosphorylated peptides may reflect the stage of dementia and may be applicable for the detection of the early stages of AD.

Studies devoted to quantitative assessment of pSer8-Aβ content mainly concern brain tissues [6,7], while analysis of the phosphorylation profile of Aβ in blood or cerebrospinal fluid (CSF) is of greater interest in the perspective of practical use as an additional parameter for diagnosing AD [8]. Recently, an electrochemical sensor conjugated with anti-Aβ40 antibodies has been developed to measure the phosphorylated-Aβ40/total-Aβ40 ratio in CSF samples from AD patients and healthy individuals [9]. The phosphorylated form averaged 10% of the total Aβ fraction and was estimated at approximately 100 pg/mL for both healthy and AD groups. Unfortunately, the available experimental data on the identification of pSer8-Aβ in CSF are scarce, so it is not possible to draw unambiguous conclusions about the p-Aβ content and its changes in the course of disease progression.

The main difficulty in the development of analytical methods for early diagnostics is the achievement of high sensitivity, since the concentrations of specific Aβ proteoforms in the biological fluids of patients are low. In addition, the most popular methods for studying the phosphoproteome involve the enrichment of phosphopeptides using metal oxide affinity chromatography or immunoassays and do not allow to measure the different proteoforms simultaneously.

MALDI-TOF MS-based methods have high sensitivity, high throughput, and speed due to the automation of the mass spectrometric analysis process, and also allow simultaneous detection of different forms of Aβ [10,11,12]. The combination of MALDI MS, with Aβ isolation by immunoprecipitation or solid phase extraction, can be further used to identify phosphorylated Aβ peptides in plasma and CSF of patients with AD. In this paper, we propose an approach for the quantitative assessment of p-Aβ based on a MALDI-TOF MS technique.

## 2. Results and Discussion

### 2.1. Optimization of Experimental Conditions for MALDI-TOF Analysis

Phosphorylation is one of the ubiquitous PTMs involved in many regulatory cellular pathways; however, correct identification of modified forms is difficult due to the lability of the phosphoryl group. Mass spectrometric analysis of pSer8-Aβ is complicated by the relatively low ionization efficiency compared with the unmodified Aβ. Thus, the study of phosphorylated peptides requires the selection of appropriate experimental conditions.

**Figure 1:**
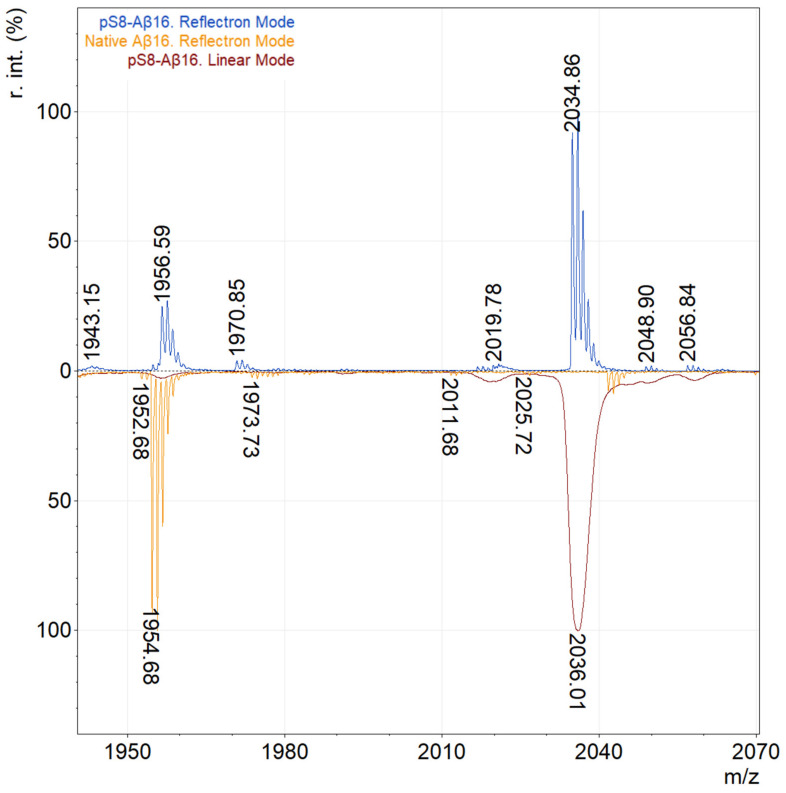
Comparison of mass spectra of pSer8-Aβ(1–16) in linear and reflectron modes. The blue line shows the mass spectrum of pSer8-Aβ(1–16) in reflectron mode, orange—Aβ(1–16) in reflectron mode, and red—pSer8-Aβ(1–16) in linear mode.

The analysis was performed in linear and reflectron modes for binary mixtures of pSer8-Aβ:Aβ (1:1) with a total concentration of 25 fmol/μL for the hydrolytic amyloid fragment 1–16 and 54 pmol/μL for the full-length peptide 1–42. In negative ion mode, the pSer8-Aβ form had a higher relative intensity than the unmodified Aβ, but the overall intensity was worse in contrast to the positive ion mode. The linear mode is more sensitive than the reflectron mode, which allows us to use the method with lower sample quantities. However, the linear mode has a low resolution, thus analytes with close *m*/*z* values can overlap with the target peak giving false positive or negative results. This limitation can be bypassed by the reflectron mode, which has a higher resolution [13]. For these reasons and in order to find out the efficiency of the method, the work was carried out in both modes. A decrease in the relative intensity of pSer8-Aβ and the appearance of new peaks overlapping with the peaks of native Aβ were observed in the reflectron mode. This can be explained by the loss of the phosphoryl group from pSer8-Aβ peptide as a result of PSD (post-source decay) during the drift to the reflectron and back [14,15,16] (Figure 1). It should be noted that no oligomers were detected in the spectra during the analysis of the samples in the entire range of the studied concentrations and matrices. This fact indicates that the possible oligomerization of Aβ, which could occur during peptides’ crystallization, does not have a noticeable effect on the method since these oligomers most likely decompose to monomers during ionization under the action of laser radiation.

**Figure 2:**
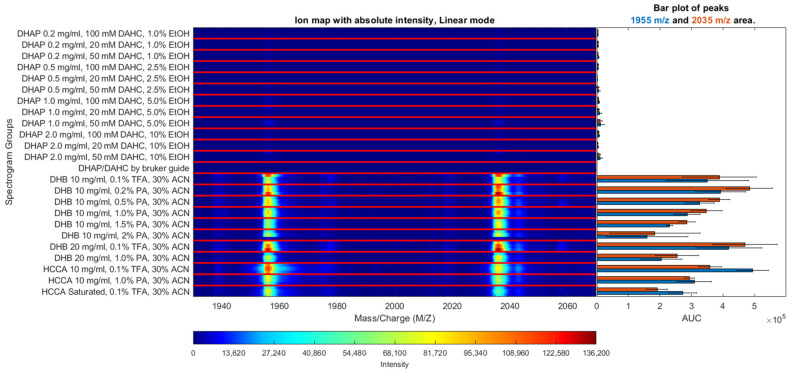
Ion map for pSer8-Aβ16 mass spectra obtained using DHAP, HCCA, and DHB matrices with the addition of various concentrations of phosphoric and trifluoroacetic acid. Aβ16 corresponds to *m*/*z* 1955; pSer8-Aβ16 corresponds to *m*/*z* 2035.

In case of MALDI-TOF MS analysis, it is critical to select a suitable matrix composition, which allows to obtain the best intensities of pSer8-Aβ and unphosphorylated Aβ, simultaneously. Three types of matrices were tried: HCCA and DHB, as the most popular for peptide ionization, and DHAP/DAHC, which demonstrated the highest efficiency in previously published experiments with phosphopeptides. Figure 2 shows ion maps for mass spectra obtained using different matrices with the addition of various concentrations of phosphoric (PA) and trifluoroacetic acid (TFA). The optimal matrix solution was determined by comparing the intensities of pSer8-Aβ and Aβ peaks. Measurements were performed for binary mixtures of the indicated proteoforms (1:1, pSer8-Aβ:Aβ).

According to our results, the highest p-Aβ intensity was observed for the DHB matrix. Although the best signal for the unmodified form was achieved using HCCA. DHAP/DAHC demonstrated a low efficiency in the case of pSer8-Aβ42 (Supplementary, Materials, Figures S2 and S3), though for pSer8-Aβ16 the results were quite satisfactory despite the low intensity of the spectral peaks in general.

Based on literary data, the effect of several phosphopeptide signal-enhancing additives were considered. Finally, the optimal matrix solutions for p-Aβ ionization were selected. The best performance (high signal-to-noise ratio as well as high intensities of target peaks) was obtained by using the DHB matrix solutions with addition of 0.1% TFA, 0.2% PA, or 0.5% PA (Figure 2 and Figure S1). Matrix solutions with PA addition demonstrated a small enhancement in intensity of p-Aβ signal compared with 0.1% TFA, which also increased the measured value of the input of p-Aβ in the sum of pSer8-Aβ and Aβ signals (in DHB up to 5% in linear mode in average) (Supplementary Materials, Figures S4–S7). However, addition of more than 0.5% of PA leads to an overall decrease in signal intensity, matrix crystallization problems, and a decrease in the number of spots with good signal intensity. Moreover, a significant disadvantage of PA addition is its incompatibility with the hydrophobic coating of AnchorChip targets, which allow to achieve additional sample concentration and thus higher sensitivity.

### 2.2. Calibration Curves

The objective of the study was to develop a highly sensitive method to estimate p-Aβ/Aβ ratio at concentrations observed in blood and CSF samples from AD patients. Practically for this purpose, calibration curves that would allow to quantify the relative amount of phosphorylated form by measuring the intensities of pSer8-Aβ and Aβ peaks were obtained.

**Figure 3:**
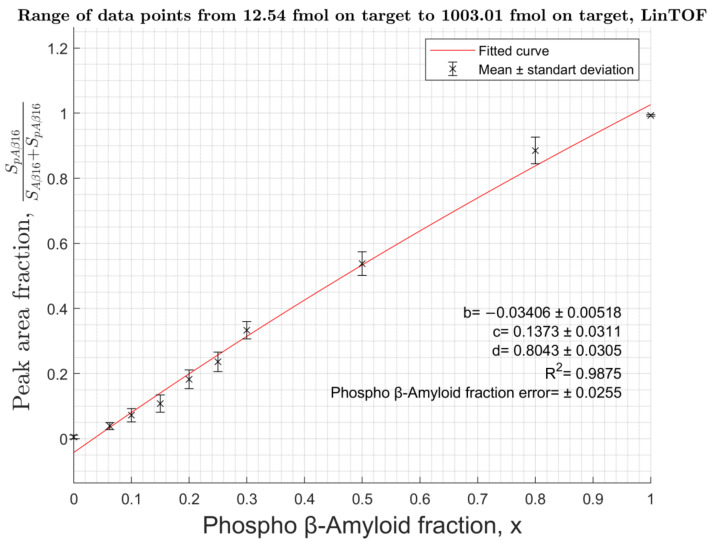
Calibration curve for Aβ16 in linear mode on an AnchorChip target with 10 mg/mL DHB, 0.1% TFA.

**Figure 4:**
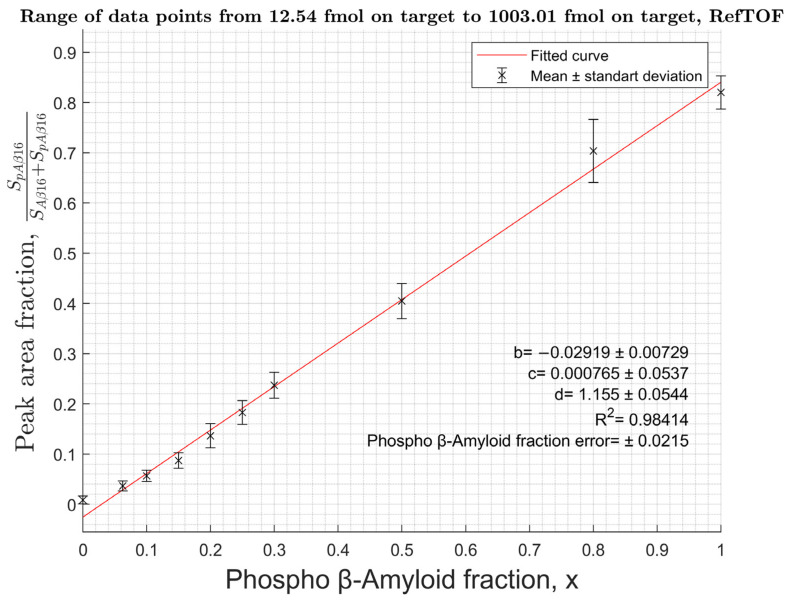
Calibration curve for Aβ16 in reflectron mode on an AnchorChip target with 10 mg/mL DHB, 0.1% TFA.

Measurements were carried out for a series of Aβ binary solutions containing pSer8-Aβ form in molar ratios from 0% to 100%. The solutions were applied to the target in an amount of 2.5 fmol to 5 pmol of total Aβ16 or from 4 pmol to 44 pmol of total Aβ42. Bruker MTP 384 polished steel target plate with a matrix solution of 20 mg/mL DHB, 0.5% PA in 30% ACN (DHB-PA), and Bruker MTP 384 AnchorChip 400 target plate with DHB 10 mg/mL, 0.1% TFA, and 30% ACN were used. To determine the intensity of the peaks corresponding to different Aβ proteoforms, the area under the isotopic cluster of the corresponding proteoform was determined. For the calibration curve, the input of the pSer8-Aβ isotope cluster to the sum of pSer8-Aβ and Aβ areas was calculated and plotted versus the fraction of pSer8-Aβ in the content of the binary mixture. The obtained curves (Figure 3, Figure 4 and Figures S8–S11) have a fractional-linear dependence and do not depend on the sample concentration. The fractional-linear character of the approximation is explained by a significant difference in the ionization efficiencies of pSer8-Aβ and the unmodified form, which leads to a significant decrease in the total signal intensity with an increase in the phosphorylated peptide content.

To obtain the theoretical equation of the curve, a statistical ionization model was used [17]. In this model, the analyte’s intensity is statistical in nature and proportional to its quantity (*η*). Each compound has its own ionization coefficient:

(1) $$I_{n}=a_{n}\times \eta_{n}$$

(2) $$I_{p}=a_{p}\times \eta_{p}$$

*I*—intensity; *α*—ionization coefficient; *η* is the amount of substance on the target; indices n—for unmodified Aβ-peptides; and *p*—for phosphorylated Aβ.

The coefficient α is considered constant for a particular analyte and independent of its total amount. For further transformations, the following variables were chosen: the relative intensity of pSer8-Aβ (*I_fr.p_*) and the relative amount of pSer8-Aβ in the analyzed sample (*x*):

(3) $$I_{fr.p}=\frac{I_{p}+I_{noise}}{I_{p}+I_{n}+2\times I_{noise}}=\frac{a_{p}\times \eta_{p}+\;I_{noise}}{a_{p}\times \eta_{p}+a_{n}\times \eta_{n}+2\times I_{noise}}\;;$$

(4) $$x=\frac{\eta_{p}}{\eta_{p}+\eta_{n}}\to \;\eta_{p}=\frac{x\times \eta_{n}}{1-x}$$

(5) $$I_{fr.p}=\frac{x+I_{noise}\times \left(1-x\right)/\left(a_{p}\times \eta_{n}\right)\;\;}{x\times \left(1-\frac{a_{n}}{a_{p}}-2\times I_{noise}/\left(a_{p}\times \eta_{n}\right)\right)+\frac{a_{n}}{a_{p}}+2\times I_{noise}/\left(a_{p}\times \eta_{n}\right)}\;;$$

where $I_{p}$—intensity of p-Aβ; $I_{n}$—intensity of unmodified Aβ, $I_{noise}$—noise intensity; $\eta_{p}$—amount of p-Aβ in the sample; and $\eta_{n}$—amount of unmodified Aβ in the sample.

Finally, a fractional-linear equation with the following theoretical coefficients was obtained:

(6) $$\frac{I_{p}}{I_{p}+I_{n}}=\frac{x+b}{x\times c+d}$$

where

(7) $$b=\frac{I_{noise}\times \left(1-x\right)}{\left(a_{p}\times \eta_{n}\right)}$$

(8) c=1−d

(9) $$d=\frac{a_{n}}{a_{p}}+\frac{2\times I_{noise}}{\left(a_{p}\times \eta_{n}\right)}\;$$

As a result, calibration curves with an accuracy of ±2% to ±4% for all Aβ proteoforms were obtained. Considering that according to the literature [9] the content of pSer8-Aβ in biological samples is estimated to be around 10% of all Aβ and its limit of detection was measured at the level of 1 fmol for the hydrolytic fragment 1–16 and 1 pmol for the pSer8-Aβ42, the working lower limit of the overall Aβ content should be approximately 10 times higher, i.e., at the level of ~10 fmol and ~10 pmol per sample, correspondingly. This limit restricts the application of the method for analysis of full-size Aβ forms due to its lower ionization, thus hydrolysis should be used to obtain the fragment 1–16 from all forms of Aβ prior to quantitative analysis. To assess the applicability of the proposed approach to biological fluids, model samples were prepared. An amount of 1 mL of diluted human serum was spiked with pSer8-Aβ42 to a final concentration of 100 ng/mL. Immunoprecipitation was performed to selectively isolate pSer8-Aβ-peptides using 1E4E11 antibodies followed by hydrolysis to obtain the Aβ16 fragment for MS analysis (Figure S12).

## 3. Materials and Methods

### 3.1. Materials

Synthetic standards of beta-amyloid 1–16 (Aβ16), beta-amyloid 1–42 (Aβ42), as well as their phosphorylated at serine 8 forms (pSer-8-Aβ16 and pSer8-Aβ42) were obtained from BioPeptide Co (San Diego, CA, USA) (Figures S13–S16). Peptides were dissolved in 10% acetonitrile. Stock solutions at a concentration of 1 mg/mL were stored at −80 °C until further use.

The following reagents were used for matrix preparation: 2,5-dihydroxybenzoic acid (DHB), α-Cyano-4-hydroxycinnamic acid (HCCA), and 2,5-Dihydroxyacetophenone (DHAP). Matrix solution additives: phosphoric acid (PA), trifluoroacetic acid (TFA), and diammonium citrate (DAHC). Solvents: acetonitrile (ACN), deionized water, and ethanol. All reagents were of HPLC/MS grade or higher and obtained from Merck KGaA (Darmstadt, Germany).

### 3.2. Selection of the Optimal Matrix for Aβ Ionization

Several matrix solutions were tested. Solutions of 10 mg/mL DHB in 30% (*v*/*v*) ACN: five samples with PA addition (ranging from 0.2–2% (*v*/*v*)) and one with 0.1% TFA. Solutions with 10 mg/mL HCCA in 30% ACN: one with 1% PA and another with 0.1% TFA.

An amount of 20 mg/mL DHAP in ethanol and 200 mM (45,236 mg/mL) DAHC were used to prepare 12 solutions with various combinations of matrix concentrations. DHAP: 0.2 mg/mL in 1% ethanol, 0.5 mg/mL in 2.5% ethanol, 1 mg/mL in 5% ethanol, 2 mg/mL in 10% ethanol—in combination with DAHC: 20 mM, 50 mM, and 100 mM (4.524 mg/mL, 11.309 mg/mL, and 22.618 mg/mL) [18].

An amount of 20 mg/mL DHB solution (1% PA, 30% ACN) was chosen as a standard for peptide ionization and the following solutions were used: DHB 20 mg/mL, 0.1% TFA, 30% ACN; HCCA saturated, 0.1% TFA, 30% ACN and DHAP 15.2 mg/mL, DAHC 20 mM (4.524 mg/mL), and 75% ethanol.

For matrix optimization experiments, a solution of p-Ser8-Aβ:Aβ (1:1) was used with a total concentration of 5 pmol/µL for the 1-16 peptide fragment and 54 pmol/µL for the 1–42 one. Solutions of pSer8-Aβ:Aβ (1:1) were applied to the Polished Steel target in 0.5 µL at three points of the target for each matrix, which was further applied in 0.5 µL after the peptide solution had dried.

### 3.3. Construction of Calibration Curves to Determine the Proportion of pS8-Aβ

Binary mixtures of phosphorylated and non-phosphorylated forms of beta-amyloid were used to build calibration curves. Total concentrations of 16-amino-acid fragments were 0.025 pmol/µL, 0.05 pmol/µL, 0.125 pmol/µL, 0.5 pmol/µL, 2.5 pmol/µL, and 5 pmol/µL, and in mixtures with 42-amino-acid fragments they were 9 pmol/µL, 13 pmol/µL, 17 pmol/µL, 21 pmol/µL, 27 pmol/µL, and 44 pmol/µL. The percentage of phosphorylated fraction in every solution varied from 0% to 100%. An amount of 1 µL of each mixture was applied to MTP 384 Polished Steel and MTP 384 AnchorChip 400 targets using the “Dried Droplet” method. Matrix formulations: 20 mg/mL DHB, 0.5% PA in 30% ACN for Polished Steel and 10 mg/mL DHB, and 0.1% TFA in 30% ACN for AnchorChip. Each binary mixture was applied to the target three times at different points. All preparations were carried out on air at room temperature.

### 3.4. Model Sample Preparation and Immunoprecipitation

To test the method performance, model samples containing p-Aβ peptides were prepared. Human serum obtained from a healthy volunteer and diluted 1:50 with PBS (pH 7.4), comparable in total protein amount with CSF, was chosen as the “matrix” solution. pSer8-Aβ solutions were prepared from a 1 mg/mL pSer8-Aβ stock dissolved in 10% acetonitrile, which was stored at −80 °C until the experiment. pSer8-Aβ42 was spiked into 1 mL of matrix solution and incubated for 1 h at room temperature. The final sample concentration was 100 ng/mL. Then, pSer8-Aβ42 was immunoprecipitated from the model samples using 1E4E11 monoclonal antibodies (Merck KGaA, Darmstadt, Germany) immobilized on Dynabeads (Thermo Fisher Scientific, Waltham, Ma, USA) according to the manufacturer’s protocols. Briefly, the model sample was spiked with 1E4E11 beads (up to ~1 μg of antibodies per sample) and mixed with a RIPA (radioimmunoprecipitation assay) buffer (final sample concentration: 50 mM HEPES, 150 mM NaCl, 0.05% SDS, 0.25% sodium deoxycholate, and 0.5% Nonidet P-40), and incubated for 1 h at room temperature. After removal of the supernatant using a magnetic rack, the beads were washed with 1 mL of 0.1% BSA/PBS solution (pH 7.4) 3 times and 1 mL of 10 mM Tris (pH 7.4) once. The target fraction was eluted using 70% ACN solution. The final samples (40 μL) were dried on a vacuum concentrator (Eppendorf, Hamburg, Germany) to the volume of 20 μL.

### 3.5. Hydrolysis

For the MALDI-TOF analysis, samples were hydrolyzed by the LysC protease (Promega, Madison, WI, USA) to obtain p-Aβ16 fragments. Samples after immunoprecipitation (20 μL) were mixed 1:1 with 0.5 μg/μL LysC prepared in 100 mM ammonium bicarbonate (up to the final volume of 40 μL) and then incubated at 37 °C for 4 h. An amount of 1 μL of a digested sample was spotted on the AnchorChip target plate and analyzed as described above. The completeness of digestion was evaluated by controlling the absence of peaks related to 1–42 peptides.

### 3.6. Mass Spectra Measurements

Experiments were conducted on an UltrafleXtreme (Bruker Daltonik Gmbh, Bremen, Germany) MALDI-TOF/TOF mass spectrometer using linear and reflectron modes in positive and negative ion modes.

Measurements for calibration curve construction were carried out in the ranges of 1900–2100 *m*/*z* for Aβ16 and 4500–4700 *m*/*z* for Aβ42. The spectra were obtained automatically by summing the signal from 5 random rasters of the target point, satisfying the condition for the presence of a peak with S/N >9.

The resulting spectra were processed using scripts and functions written in MATLAB R2021b with the Bioinformatics Toolbox application. The proportion of p-Ser8-Aβ intensity was calculated from the obtained intensity values.

## Data Availability

Data related to this study is available from the corresponding author upon reasonable request.

## Supplementary Materials

The following supporting information can be downloaded at: https://www.mdpi.com/article/10.3390/molecules27238406/s1. Figure S1–S3: Ion map for mass spectra; Figure S4–S7: Comparison of the areas under the curves corresponding; Figure S8–S11: plotted versus the fraction of pSer8-Aβ in the content of the binary mixture; Figure S12: Mass spectrum of the hydrolyzate of the beta-amyloid fraction obtained from the model sample; Figure S13–S16: beta-amyloid 1–16 (Aβ16), beta-amyloid 1–42 (Aβ42), as well as their phosphorylated at serine 8 forms (pSer-8-Aβ16 and pSer8-Aβ42).
