# Supplementary material for: Quantitative Assessment of Serine-8 Phosphorylated β-Amyloid Using MALDI-TOF Mass Spectrometry
Source: Molecules. 2022 Dec 1;27(23):8406. doi: 10.3390/molecules27238406 (PMC9740911; doi:10.3390/molecules27238406)
Supplement: Supplementary file 1 [file molecules-27-08406-s001.zip › molecules-2039206-supplementary.pdf]

Supporting Information

Article

Quantitative Assessment of Serine-8 Phosphorylated β-amyloid using MALDI-TOF Mass Spectrometry

Andrey Kuzin; Galina S. Stupnikova; Polina A. Strelnikova; Ksenia V. Danichkina; Maria I. Indeykina; Stanislav I. Pekov*; Igor A. Popov*

....

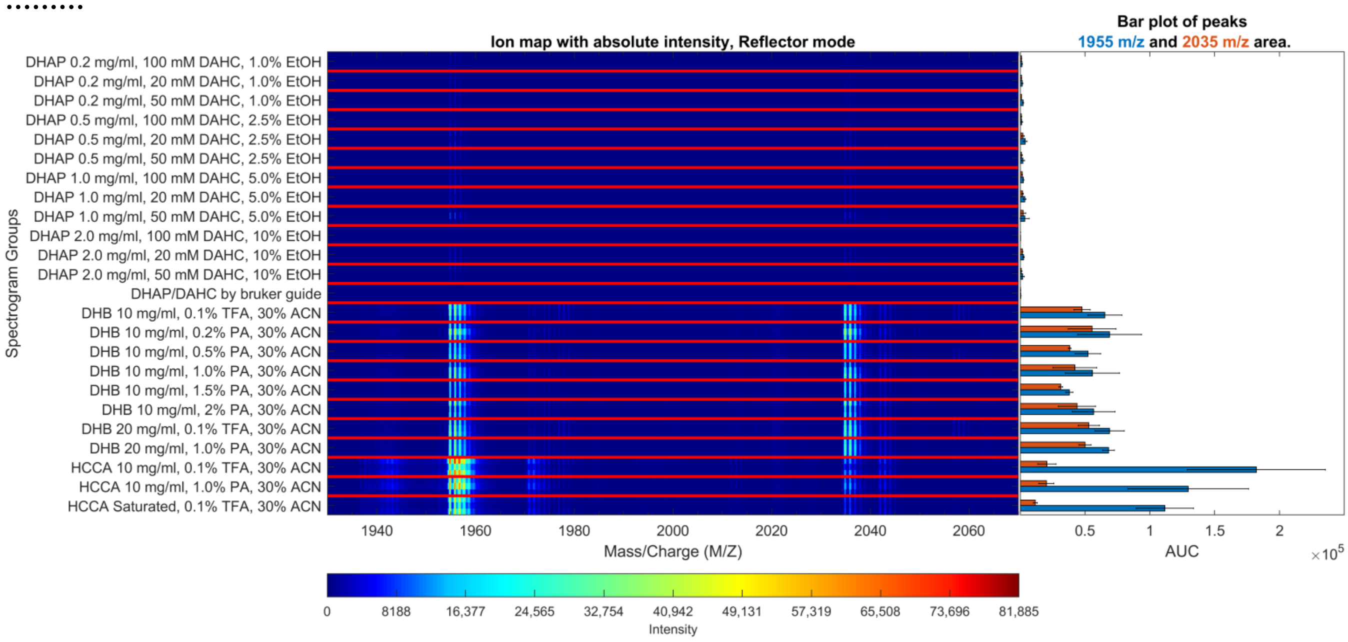

....

Figure S1. Ion map for pSer8- Aβ16 mass spectra obtained using different matrices with the addition of various concentrations of phosphoric and trifluoroacetic acid in reflectron mode. Aβ16 corresponds to m/z 1955, pSer8- Aβ16 corresponds to m/z 2035.

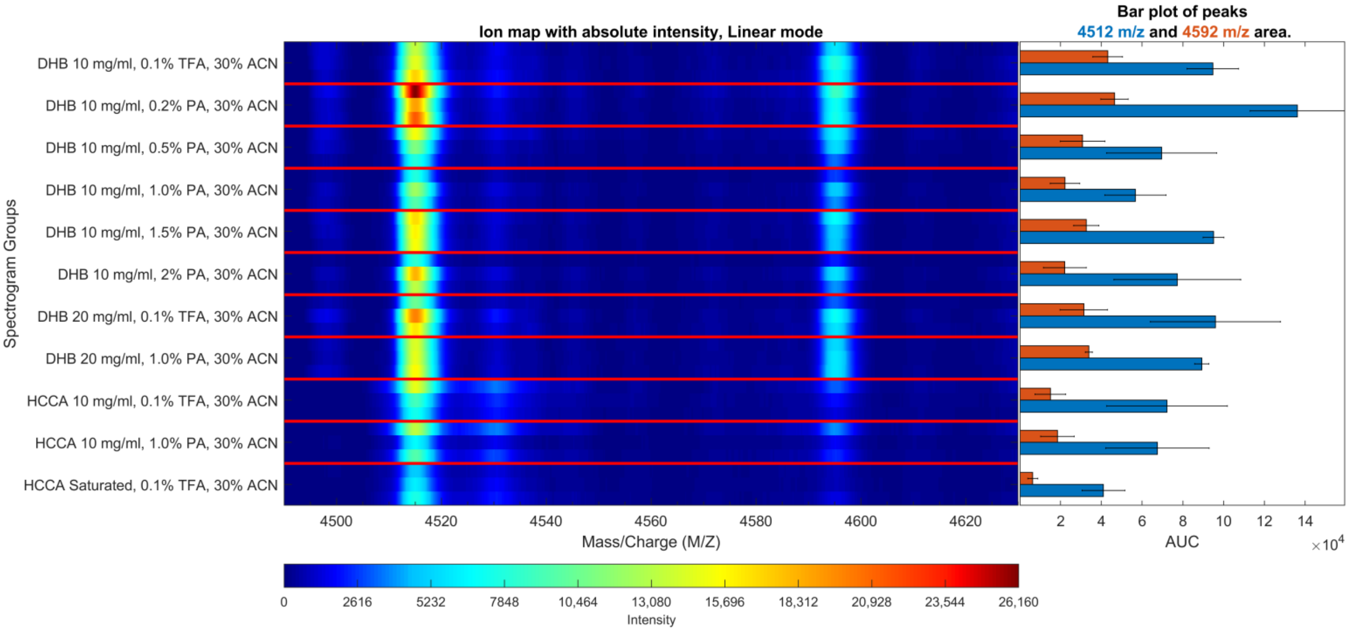

Figure S2. Ion map for pSer8- Aβ42 mass spectra obtained using DHB and HCCA matrices with the addition of various concentrations of phosphoric and trifluoroacetic acid in linear mode. Aβ42 corresponds to m/z 4512, pSer8- Aβ42 corresponds to m/z 4592.

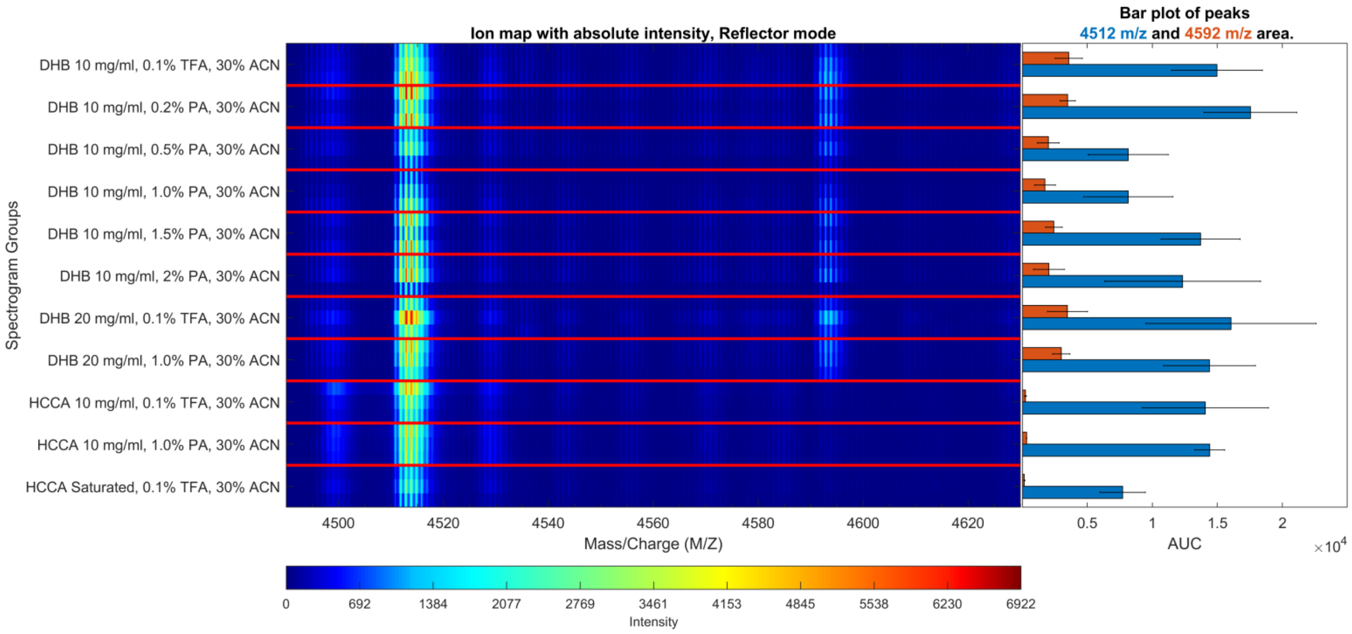

Figure S3. Ion map for pSer8- Aβ42 mass spectra obtained using DHB and HCCA matrices with the addition of various concentrations of phosphoric and trifluoroacetic acid in reflectron. Aβ42 corresponds to m/z 4512, pSer8- Aβ42 corresponds to m/z 4592.

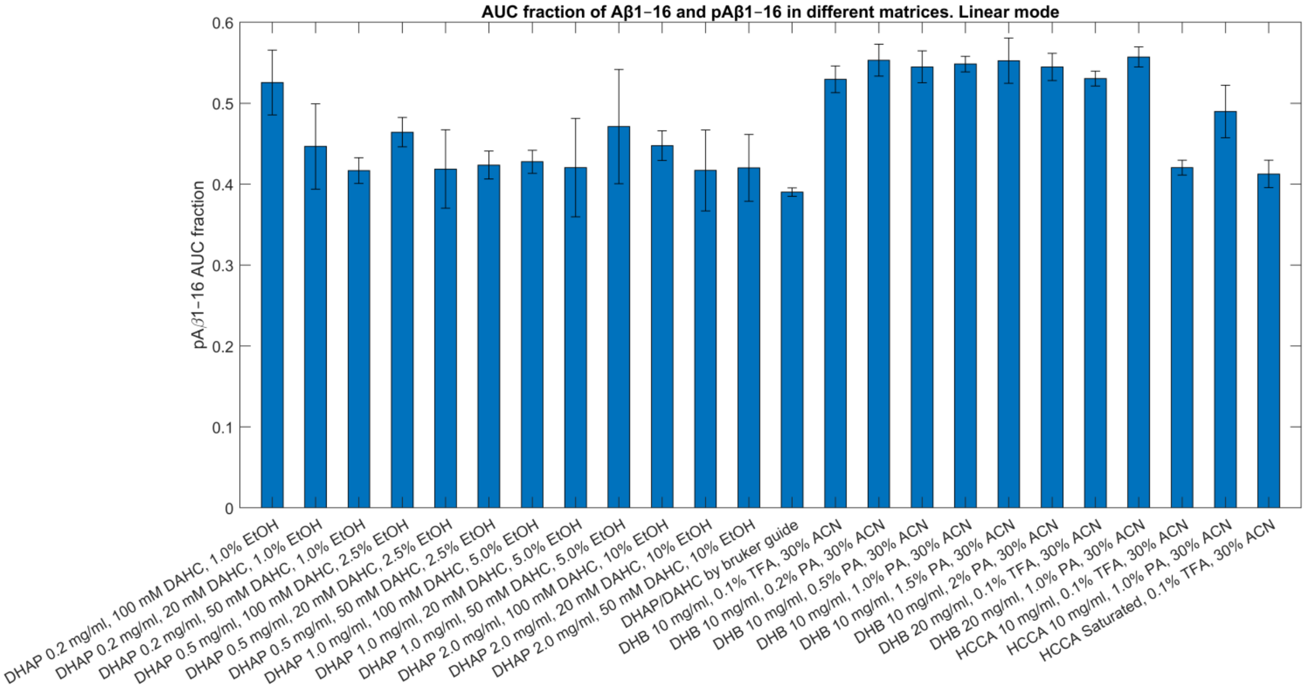

Figure S4. Comparison of the areas under the curves corresponding to pSer8-Aβ16 and Aβ peaks using different matrix solutions using linear mode.

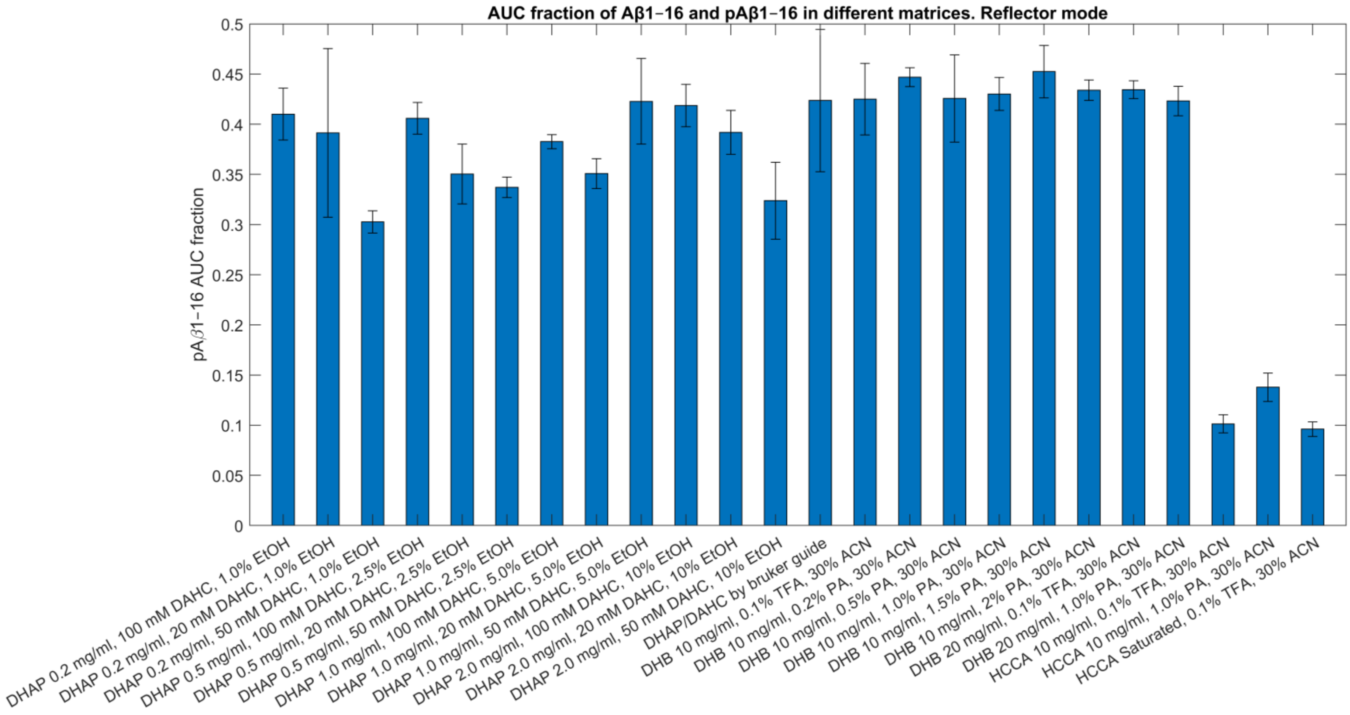

Figure S5. Comparison of the areas under the curves corresponding to pSer8-Aβ16 and Aβ peaks using different matrix solutions using reflectron mode.

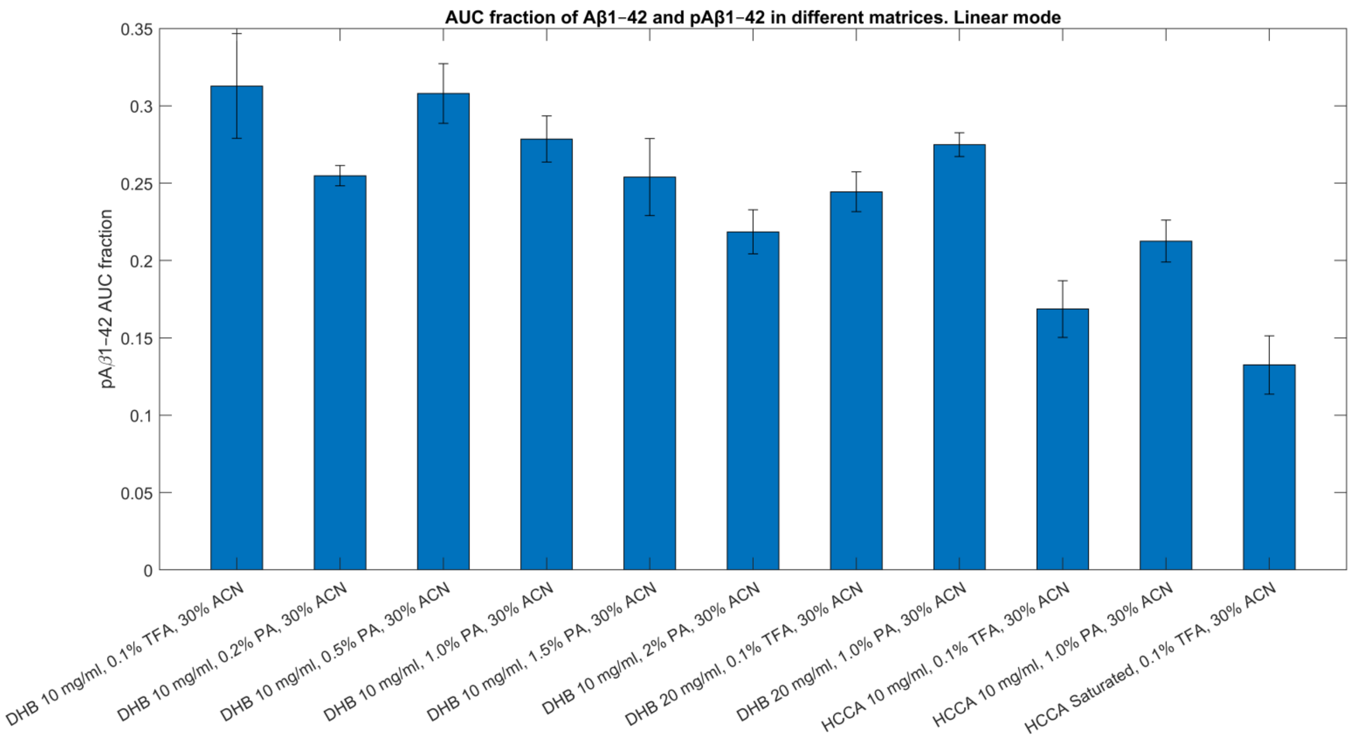

Figure S6. Comparison of the areas under the curves corresponding to pSer8-Aβ42 and Aβ peaks using different matrix solutions using linear mode.

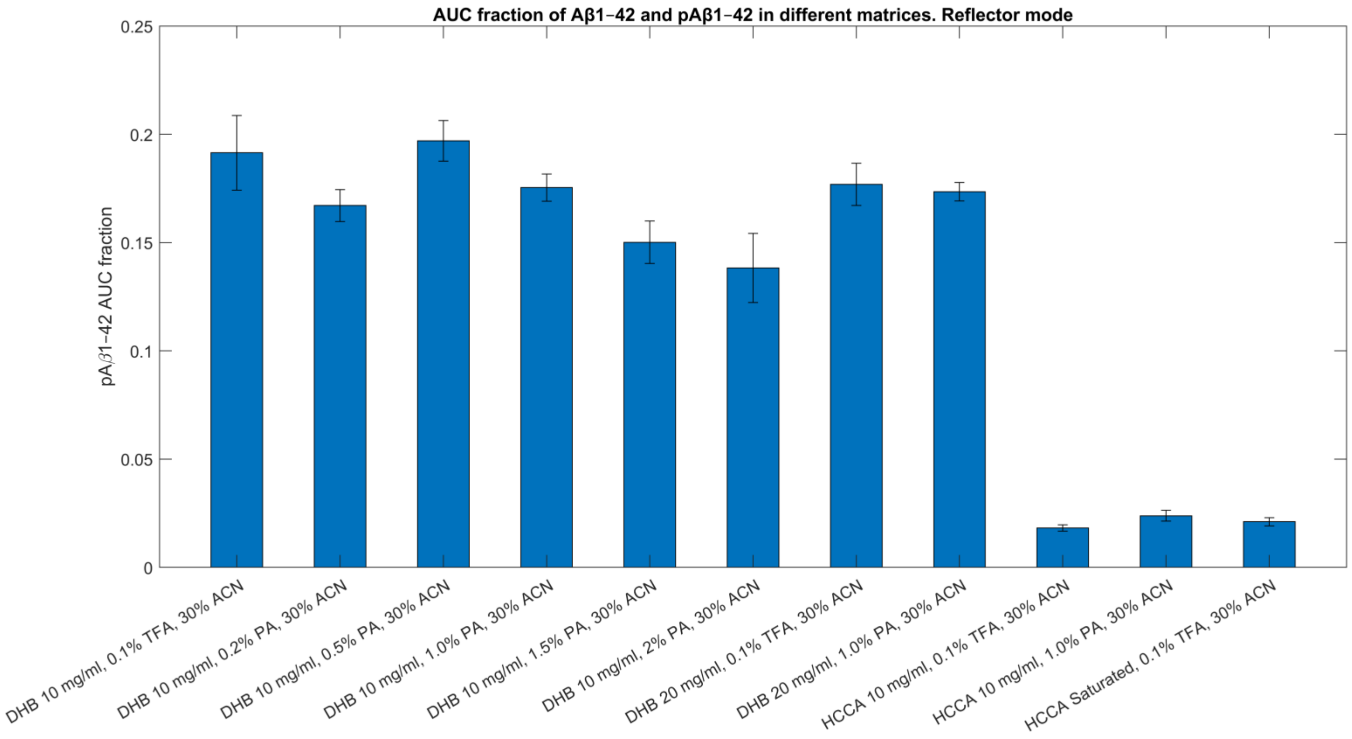

Figure S7. Comparison of the areas under the curves corresponding to pSer8-Aβ42 and Aβ peaks using different matrix solutions using reflectron mode.

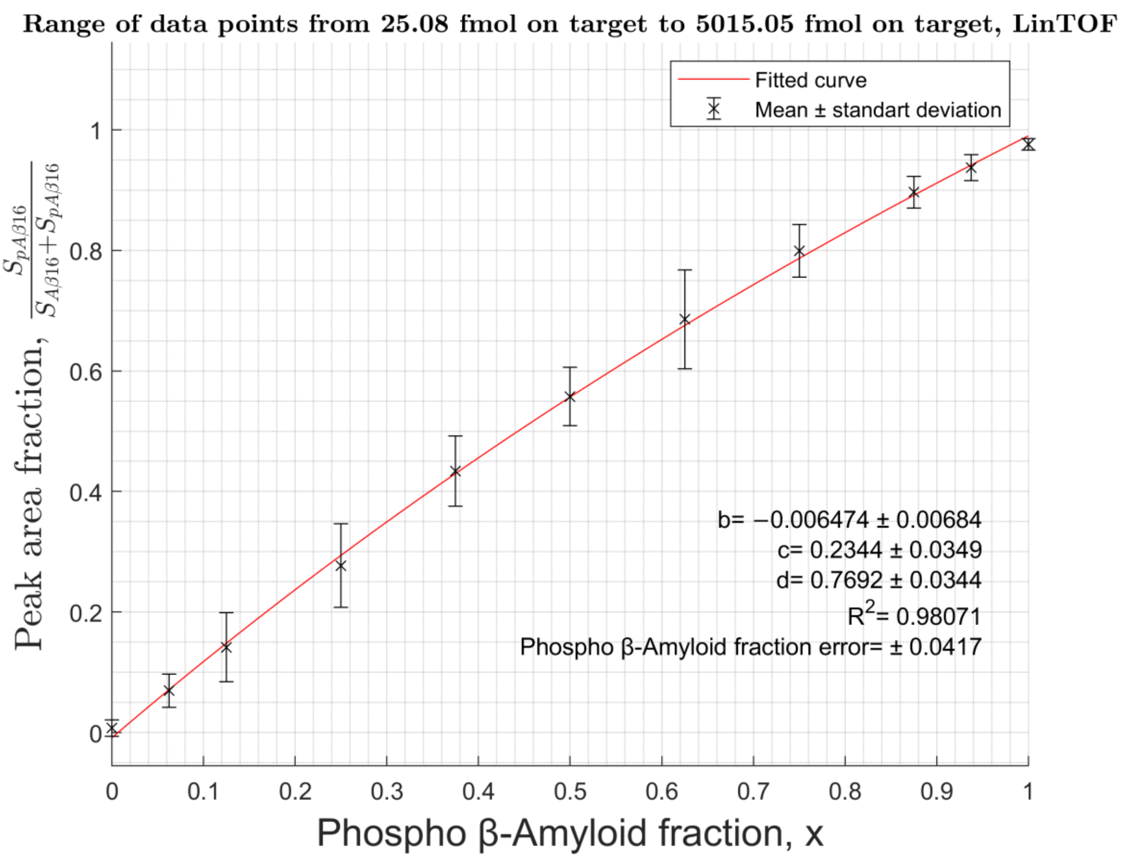

Figure S8. Calibration curve for Aβ16 in linear mode on a Polished Steel target, 10 mg/ml DHB, 0.5% PA.

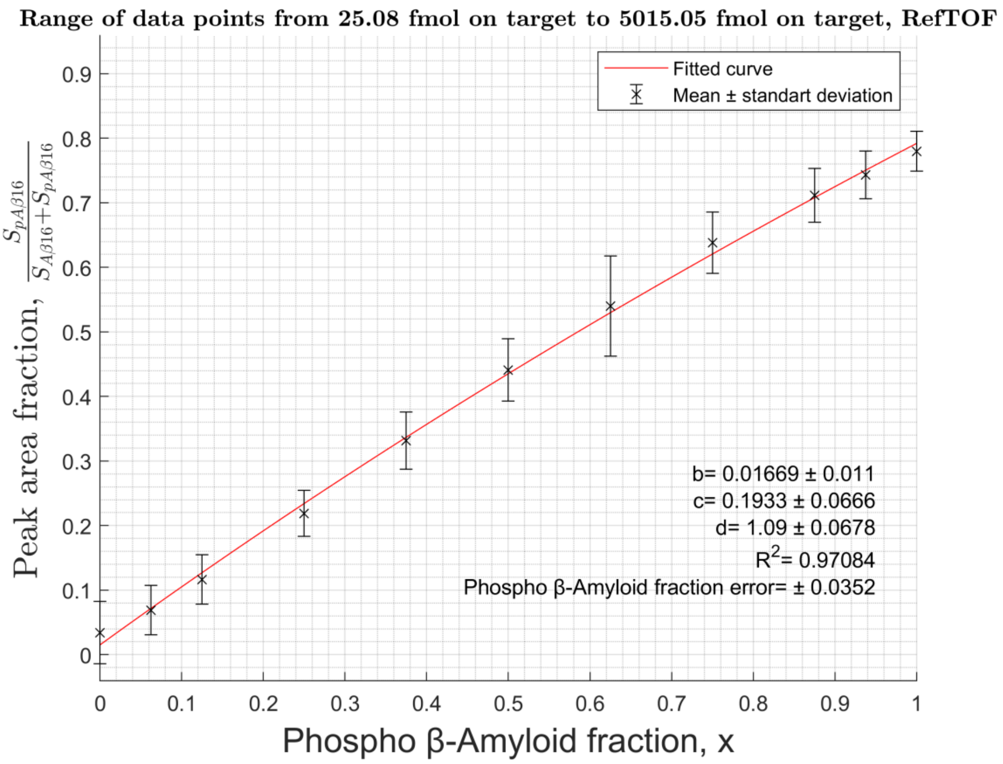

Figure S9.Calibration curve for Aβ16 in reflectron mode on a Polished Steel target, 10 mg/ml DHB, 0.5% PA.

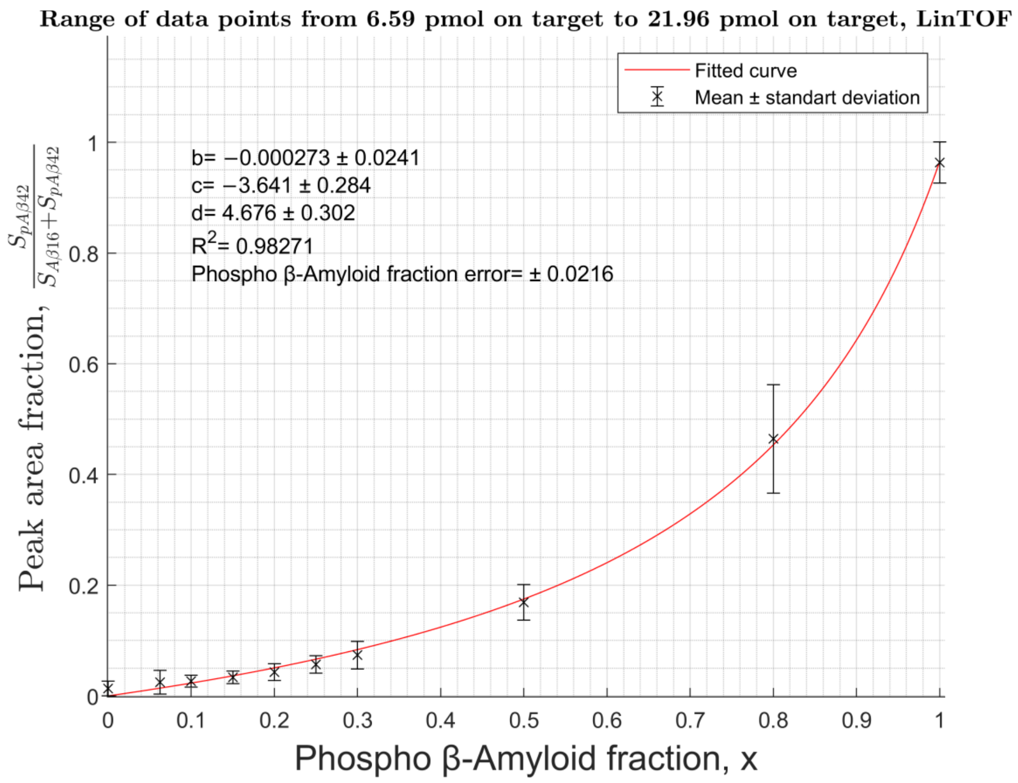

Figure S10.Calibration curve for Aβ42 in linear mode on an AnchorChip target, 10 mg/ml DHB, 0.1% TFA.

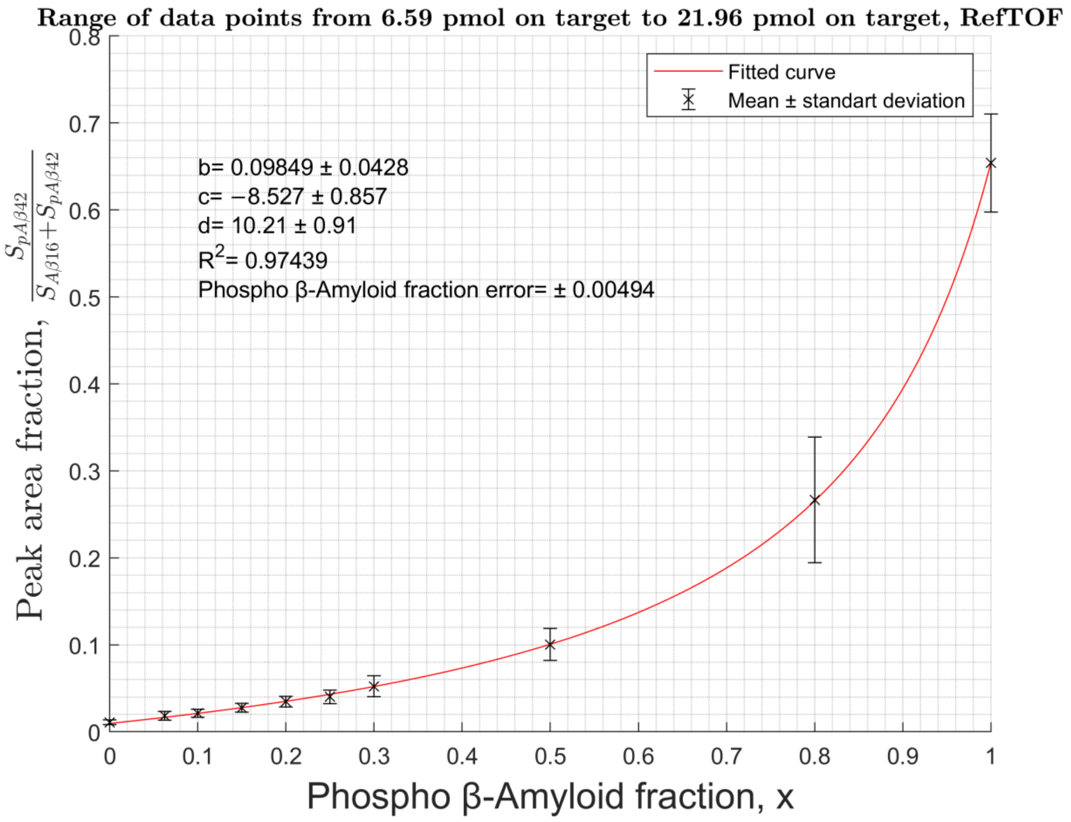

Figure S11.Calibration curve for Aβ42 in reflectron mode on an AnchorChip target, 10 mg/ml DHB, 0.1% TFA.

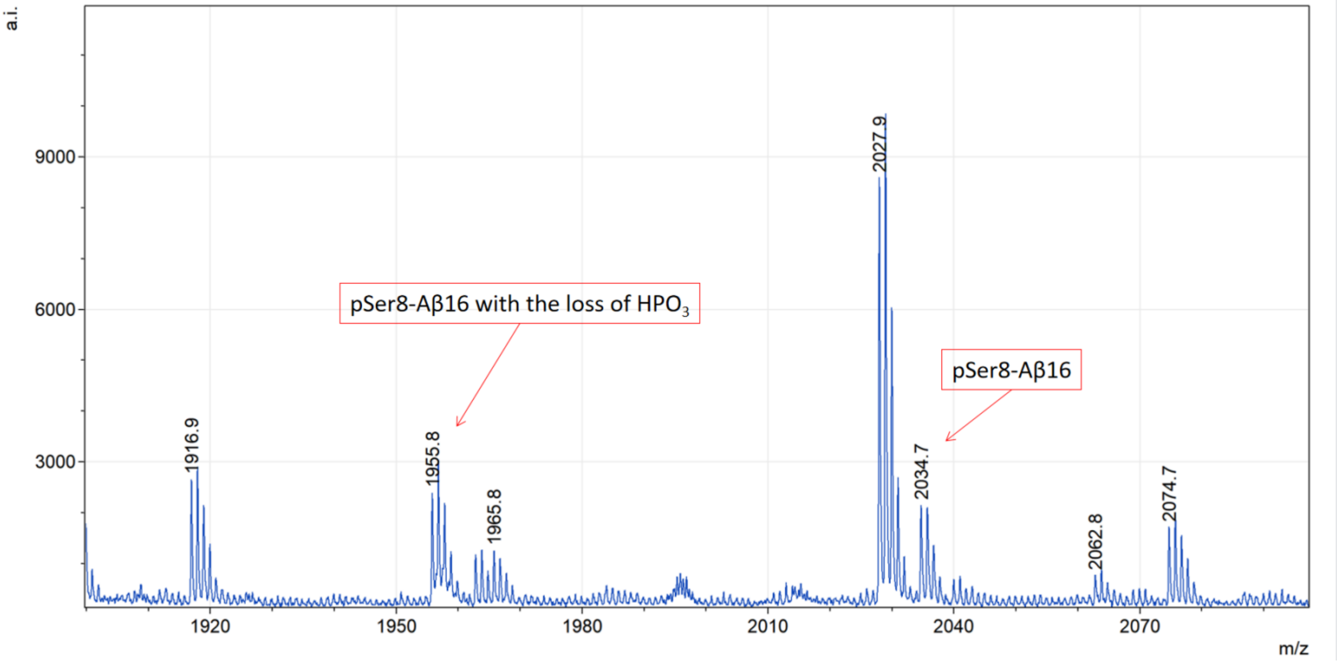

Figure S12. Mass spectrum of the hydrolyzate of the beta-amyloid fraction obtained from the model sample.

Human serum diluted 1:50 was spiked with pSer8-Aβ42 to a final concentration of 100 ng/mL, after which hydrolysis was performed to obtain pSer8-Aβ16 fragments.

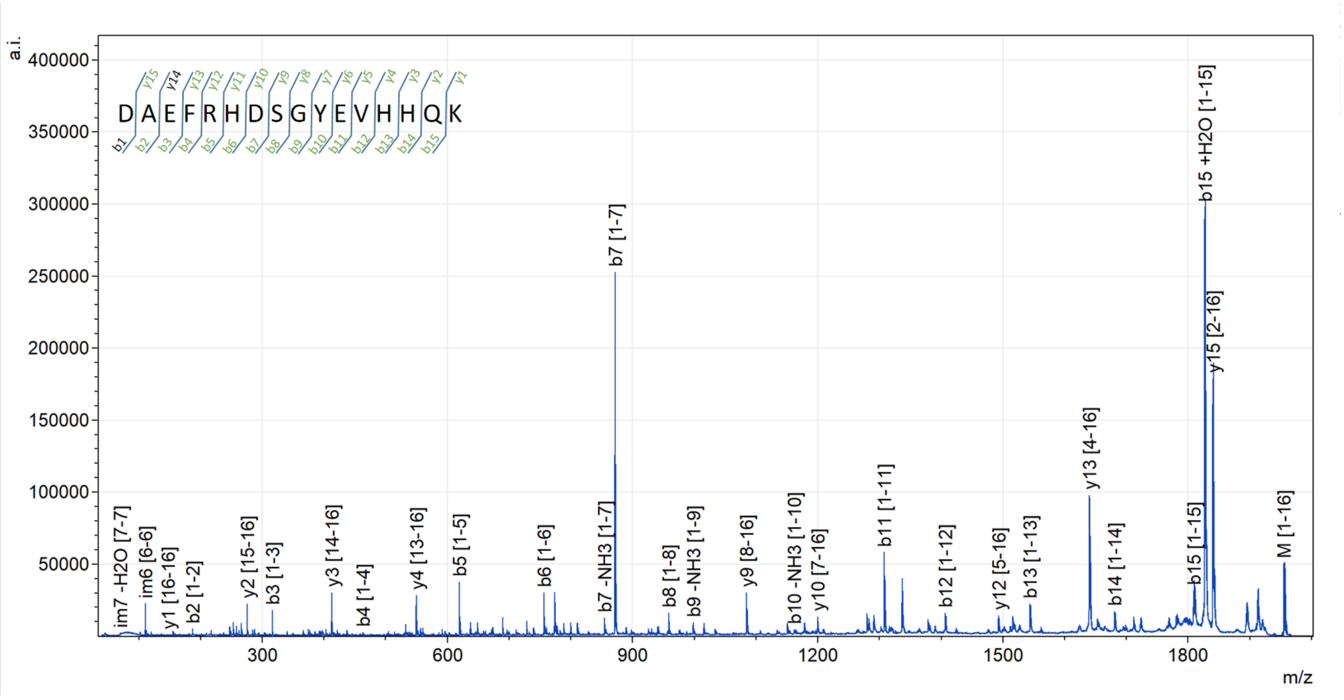

Figure S13. MS/MS of Aβ16 from BioPeptide Co. Fragments in peptide sequence marked with color if their peak in spectra with the same m/z: green - finded; black - not finded.

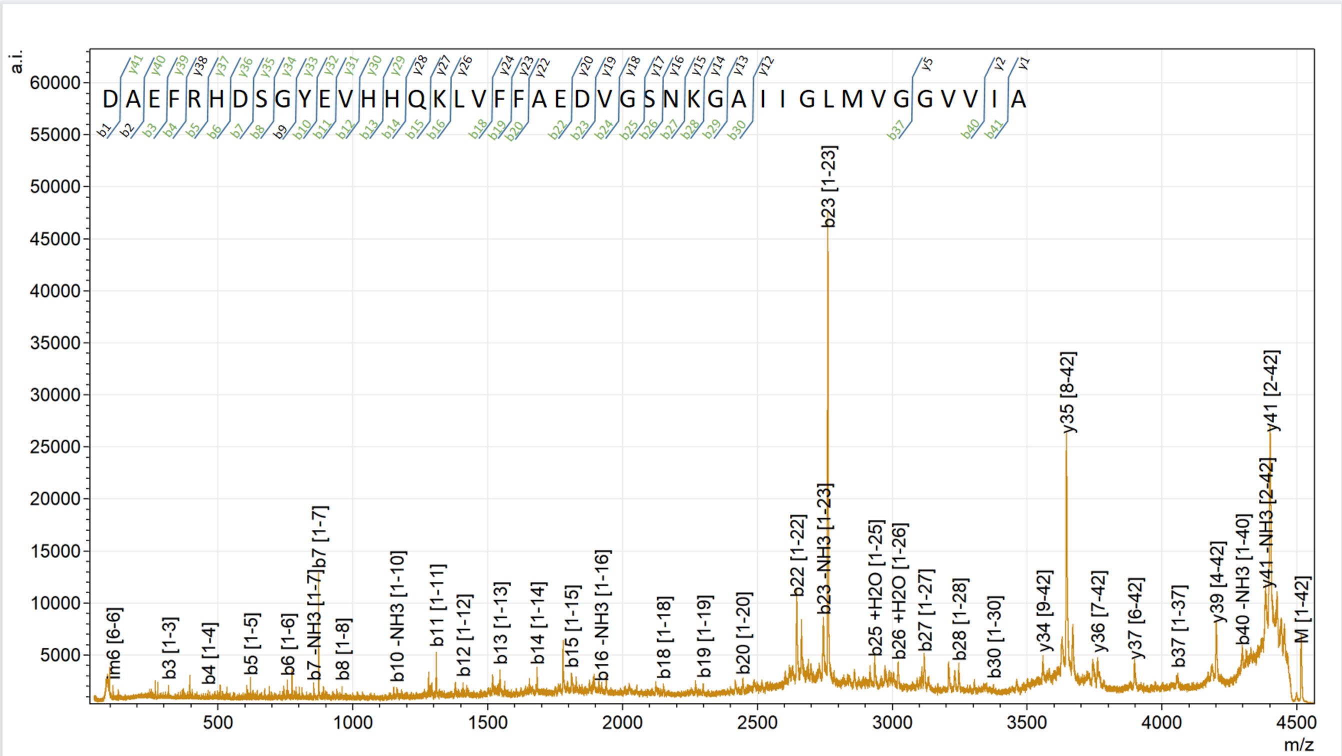

Figure S14. MS/MS of Aβ42 from BioPeptide Co. Fragments in peptide sequence marked with color if their peak in spectra with the same m/z: found - green; not found - black.

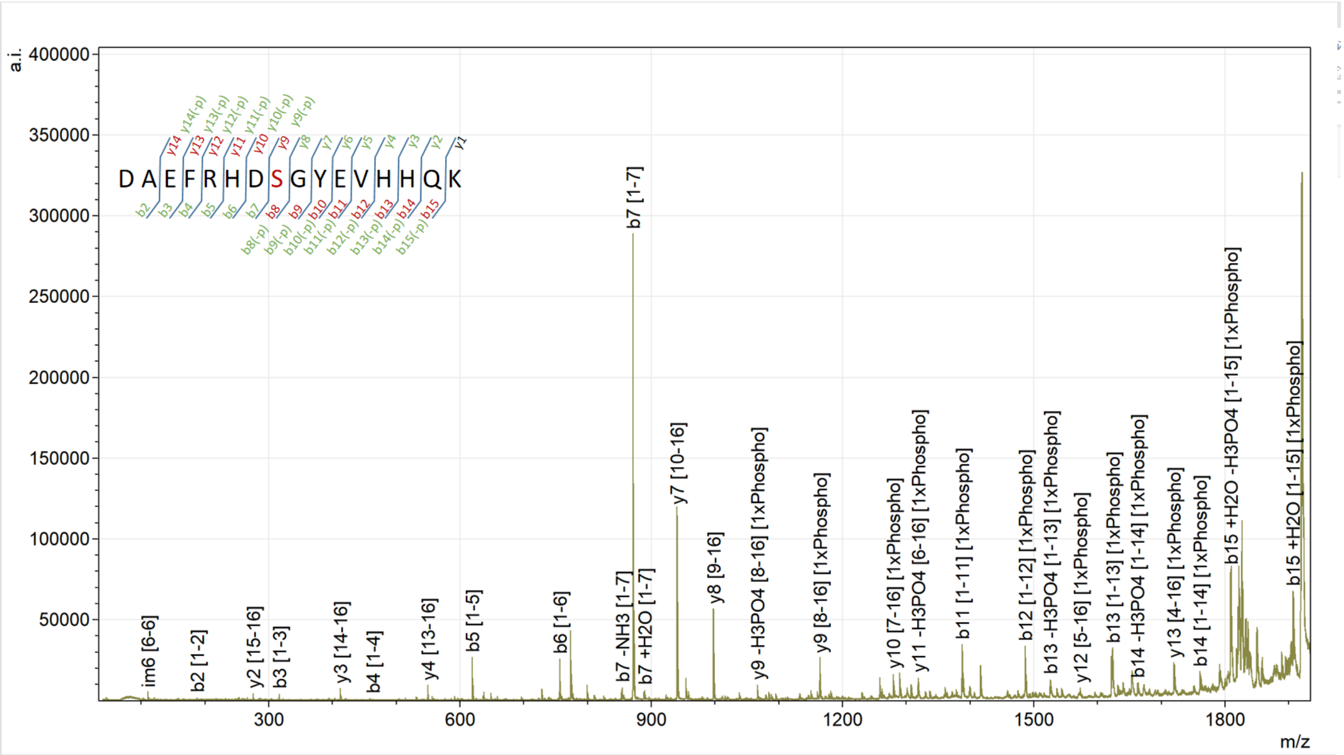

Figure S15. MS/MS of pAβ16 from BioPeptide Co. Fragments in peptide sequence marked with color if their peak in spectra with the same m/z: detected and have phospho group - red; found and it doesn't have phospho group or has lost it - green; not found - black.

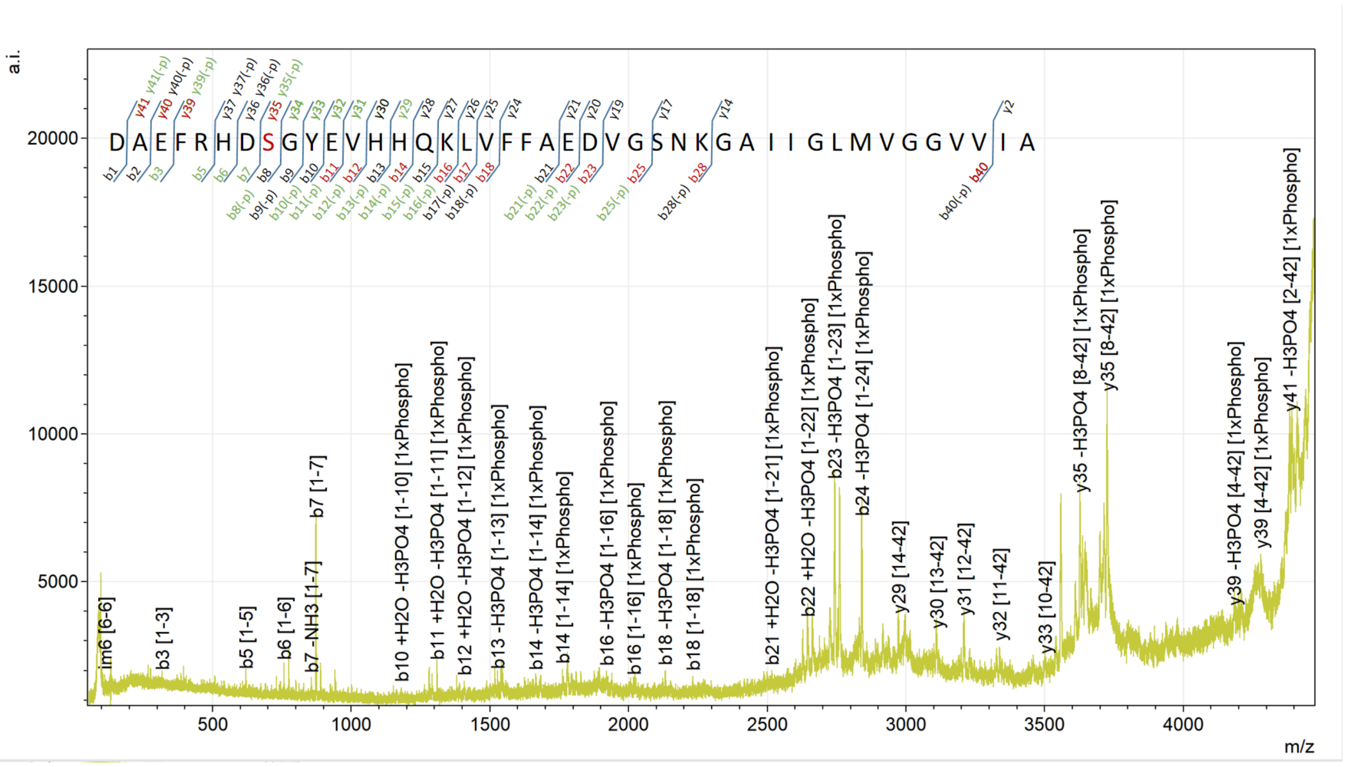

Figure S16. MS/MS of Aβ42 from BioPeptide Co. Fragments in peptide sequence marked with color if their peak in spectra with the same m/z: detected and have phospho group - red; found and it doesn’t have phospho group or has lost it - green; not found - black.
